# Efficacy of Neoadjuvant Targeted Therapy in Treatment of Patients with Localised Clear-Cell Renal Cell Carcinoma

**DOI:** 10.1155/2021/6674637

**Published:** 2021-04-30

**Authors:** O. A. Voylenko, O. E. Stakhovsky, I. V. Vitruk, O. A. Kononenko, M. V. Pikul, S. L. Semko, E. O. Stakhovsky

**Affiliations:** ^1^Senior Researcher of Department of Plastic and Reconstructive Oncological Urology, National Cancer Institute, 33/43 Lomonosova Str., Kyiv 03022, Ukraine; ^2^Researcher of Department of Plastic and Reconstructive Oncological Urology, National Cancer Institute, 33/43 Lomonosova Str., Kyiv 03022, Ukraine; ^3^Urologist of Department of Plastic and Reconstructive Oncological Urology, National Cancer Institute, 33/43 Lomonosova Str., Kyiv 03022, Ukraine; ^4^Head of Department of Plastic and Reconstructive Oncological Urology, National Cancer Institute, 33/43 Lomonosova Str., Kyiv 03022, Ukraine

## Abstract

**Aim:**

This study aimed to evaluate the efficacy of neoadjuvant targeted therapy (TT) in patients with localised clear-cell renal cell carcinoma (RCC).

**Materials and Methods:**

A special randomised trial was planned and conducted by the Research Department of Plastic and Reconstructive Oncology in the National Cancer Institute of Ukraine for testing the clinical efficacy of neoadjuvant TT in the treatment of clear-cell localised RCC, and the primary endpoint was tumour response evaluation after TT. The secondary endpoints included evaluation of dependence between the use of neoadjuvant TT and the probability of partial nephrectomy and the correlation between tumour size, stage, remaining functioning parenchyma volume, and response to systemic therapy.

**Results:**

Overall, 118 patients met the inclusion criteria and were randomly assigned to receive combined treatment or surgery alone. The percentage of tumour regression ranged from 0% to 60%, and the median was (95% confidence interval) 20.5 ± 14.3 (16.8–24.3%). Most of the patients had a slightly positive response to TT (3%–29% decrease in tumour size); *n* = 44 (76.9%) cases. Partial response by the Response Evaluation Criteria in Solid Tumours, version 1.1, was observed in 14 (24.1%) patients and reached a maximum of 60% regression. Tumour reduction in the neoadjuvant TT group allowed kidney preservation in 53 (91.4%) patients. In the control group, the number of organ-sparing procedures was significantly lower (*n* = 20, 33.3%). The statistical difference was relevant (*x*^2^ = 42.1; *p* < 0.001).

**Conclusion:**

The positive results of neoadjuvant TT obtained in our study indicate the clinical validity of combined treatment in patients with localised RCC.

## 1. Introduction

Partial nephrectomy is considered to be the surgery of choice in the treatment of localised renal cell carcinoma (RCC) [1, 2], and its oncological and functional efficacy have been proven in numerous clinical trials. However, the indications for this organ-sparing surgery have not been clearly determined.

Radical nephrectomy is commonly used in clinical practice in cases of renal hilum tumours or large tumours (>4 cm) and significantly impairs patient survival due to a decrease in filtration rate and quality of life [3–6]. In such cases, tumour size reduction can prompt kidney preservation, resulting in significant preferences for the patient. This goal can be achieved using neoadjuvant targeted therapy (TT).

According to modern literature analysis, neoadjuvant TT in localised RCC can be used only in elective clinical cases. The current data suggest performing it only during imperative indications (single kidney tumour, bilateral kidney tumours, pathology in the opposite kidney, significantly affecting its function) [7–12], and there are no randomised trials concerning the use of TT for the treatment of localised RCC.

## 2. Patients and Methods

### 2.1. Study Design

**Figure 1:**
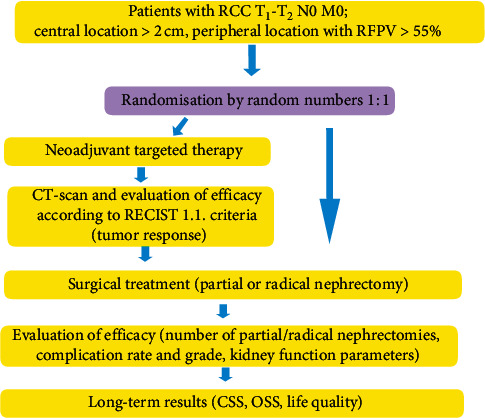
Efficacy of neoadjuvant targeted therapy in treatment of patients with localised clear-cell renal cell carcinoma study flowchart.

A special randomised trial was planned and conducted by the Research Department of Plastic and Reconstructive Oncology at the National Cancer Institute of Ukraine, testing the clinical efficacy of neoadjuvant TT in the treatment of clear-cell localised RCC. The eligibility of participating surgeons included sufficient surgical experience of >100 partial/radical nephrectomies per surgeon annually. The study was approved by the Institutional Review Board and local ethics committee, which was conducted as per with the Declaration of Helsinki and the Good Clinical Practice guidelines. The study flowchart is shown in Figure 1.

### 2.2. Patients

The inclusion criteria were clinically and histologically confirmed localised clear-cell RCC (ccRCC) (T1-T2 N0 M0) with intermediate indications for both radical and partial nephrectomy:

1. Lesion larger than 20 mm located in the renal hilum
2. Peripheral or polar RCC extending to kidney sinus
3. Remaining functioning parenchyma volume (RFPV) over 55%

Patients with chronic kidney disease, expected estimated glomerular filtration rate (eGFR) > 30 mL/min after surgery, coexisting malignant disease, and a history of any other systemic therapy were excluded. It was mandatory to exclude tumour extension and metastatic disease before patient enrolment. A special informed consent form was obtained from the study team and signed along with the National Health Care system form during the investigation. Tumour biopsy to confirm ccRCC was performed routinely, not less than 2 weeks before randomisation.

### 2.3. Randomisation and Treatment

Patients were randomly assigned in a 1 : 1 ratio to receive either a combination of neoadjuvant TT with surgery (main group) or surgery alone (control group). Randomisation was performed using a random number generation method at the randomisation centre of the National Cancer Institute.

TT in the main group was performed according to the standard schedule with drugs provided by the National Health Care System: pazopanib 800 mg daily orally for two cycles (one cycle equals 4 weeks of intake) or sunitinib 50 mg daily for two cycles (one cycle equals 4 weeks of intake/2 weeks washout interval). Patients were informed by the investigators about the side effects and oncological efficacy of both drugs before signing the informed consent form and choosing precise medication. In cases of AE greater than level 2, dose reduction was planned, with interruption in patients who might have had life-threatening conditions. Patient safety was the primary goal of the study.

Surgical treatment of the main group was planned to start from 2 weeks after neoadjuvant TT cessation or termination for complete excretion of the target drug from the body for better tissue regeneration after surgical treatment. Patients in the control group underwent surgery for 14 days after randomisation. Preference was given for partial nephrectomy in both the groups. The surgical strategy was discussed on a multidisciplinary board. The main indication for kidney preservation was an RFPV over 55%.

Treatment efficacy was evaluated based on imaging methods (computed tomography (CT) and magnetic resonance imaging), which were used in the initial examination and in all control examinations. The primary tumour was evaluated using the Response Evaluation Criteria in Solid Tumours, version 1.1 (RECIST) [13]. In the main group, kidney lesions were assessed as target lesions following response evaluation after two cycles of TT according to imaging data.

### 2.4. Follow-Up

According to the standard of care, the patients did not receive any adjuvant therapy. Follow-up examinations, including CT, were performed every 6 months up to 2 years postoperatively. Thereafter, it was performed once a year.

### 2.5. Outcomes

The primary endpoint was tumour response evaluation after TT. The secondary endpoints included evaluation of dependence between the use of neoadjuvant TT and the probability of partial nephrectomy and correlation between tumour size, RFPV, and response to systemic therapy. Recurrence-free survival, cancer-specific survival, and overall survival were not reached at the period of data cutoff.

### 2.6. Statistical Analysis

The results were statistically analysed using SPSS (version 24.0) software. The distribution of continuous data in the group was estimated using the Kolmogorov–Smirnov criterion. Descriptive statistics included calculating the median with a standard deviation or median of 25–75 percentiles. Quantitative comparisons between groups were conducted using the Mann–Whitney criterion and qualitative comparisons using the Pearson test. A total statistical estimation of regression was performed using the analysis of variance (ANOVA) test. Type I errors less than 5% (*p* < 0.05) were considered to be statistically significant differences.

## 3. Results

### 3.1. Patients and Treatment

Patients were enrolled between January 2017 and October 2018. Overall, 118 patients met the inclusion criteria and were randomly assigned to receive combined treatment or surgery alone. TT was performed in 58 (49%) patients, whereas surgery alone was performed in 60 (51%) patients.

Information on baseline patient characteristics, tumour stage and size, kidney function, and concomitant pathology are summarised in Table 1. As shown in Table 1, no statistical difference between baseline characteristics was found between the groups at randomisation.

It should be noted that about half of the patients (*n* = 57; 48%) had concomitant pathology in other organs and systems, which affected kidney function both in the preoperative and postoperative periods. The most frequent complications were hypertension in 47 (39.8%) patients, obesity in 41 (34.7%), contralateral kidney cysts in 14 (11.9%), urinary stone disease in 11 (9.3%), diabetes in five (4.2%), contralateral kidney hypoplasia in two (1.7%), and a history of myocardial infarction in three (2.5%) patients.

### 3.2. Clinical Outcome

**Figure 2:**
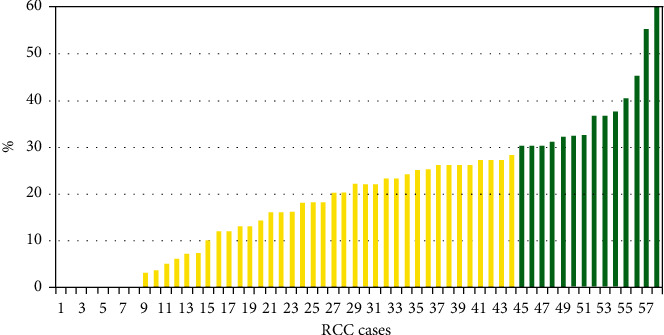
Percentage of regression of localised RCC after 2 cycles of neoadjuvant TT according to the results of spiral CT with bolus contrast enhancement, *n* = 58. Stable disease according to RECIST 1.1 (up to 30% regression) is shown as yellow, and partial regression according to RECIST 1.1 (30% regression to 100%), green. RCC, renal cell carcinoma; CT, computed tomography; TT, targeted therapy; RECIST, Response Evaluation Criteria in Solid Tumours.

According to the primary endpoint, we estimated the percentage of tumour regression in 58 patients in the neoadjuvant TT group. The degree of tumour regression in each case is shown in Figure 2. The results showed that tumour size in the prevalence of cases decreased after neoadjuvant TT. Thus, the percentage of the tumour regression ranged from 0% to 60% and the median was (95% confidence interval (CI)) 20.5 ± 14.3 (16.8–24.3%). Patients who underwent neoadjuvant TT had no evidence of disease progression. No response to targeted therapy (0% regression) was observed in eight (13.8%) cases. Thus, most of the patients had a slight positive response to TT (3%–29% of tumour size decrease); *n* = 44 (76.9%) cases. A partial response was observed in 14 patients (24.1%) and reached a maximum of 60% regression. No complete response events were observed in the study cohort. After two blocks of neoadjuvant TT in patients with localised RCC, the mean kidney tumour size reduction was 12.3 mm from (M ± SD (95% CI)) 60.8 ± 19.7 (55.7–66) to 48.5 ± 16.4 (44.2–52.8) mm (*t*-test; *p* < 0.001), which could have influenced surgical tumour complexity.

We performed a comparative analysis of RFPV before and after neoadjuvant TT in patients with localised RCC. The use of neoadjuvant TT in patients with localised RCC (Mann–Whitney *U* test; *p* < 0.001) increased the median (Me) RFPV by 21% from (Me [25%; 75%]) 62 (57; 77) mm to 83 (70; 90) mm. (Additional information is provided in Supplementary Materials.)

The distribution of partial and radical nephrectomies in the study groups is shown in Table 2. Provided with the same baseline clinical and nephrometric parameters in groups of comparison before treatment, tumour reduction seen in the neoadjuvant TT group allowed kidney preservation in 53 cases (91.4%). In the control group, the number of organ-sparing procedures was significantly lower, accounting for only 20 (33.3%). The statistical difference was relevant (*x*^2^ = 42.1; *p* < 0.001).

The correlation between tumour size before and after neoadjuvant TT using Pearson correlation analysis revealed a relevant direct correlation (*r* = 0.77613; *p* < 0.001) of the tumour size before neoadjuvant treatment on the expected outcome. (Additional information is provided in Supplementary Materials.)

Furthermore, we analysed the dependence between the objective response level and the size of the primary lesion. The data indicated that there was no direct correlation between the tumour objective response level and tumour size. Thus, for a 113 mm tumour, the objective response rate was 43%, while another large tumour (93 mm) did not respond to neoadjuvant TT at all (0% regression). Similar differences in objective responses were observed in small tumours. (Additional information is provided in Supplementary Materials.)

The evaluation was also performed in terms of comparing the T-stage and regression. Total statistical estimation performed using the ANOVA test revealed no influence of stage on tumour regression. However, the effect *η*^2^ = 0.01, with an impact power of 0.1, was not significant (*p*=0.72). Post hoc analysis using the Newman–Keuls test showed no significant difference between all subgroups of the study. Thus, between T1a and T1b, the confidence indicator was *p*=0.8 (between groups T1b and T2; *p*=0.6). Therefore, we have not confirmed the theory of greater efficacy of TT in patients with primary small RCC [14].

### 3.3. Adverse Events and Complication Rates

Adverse events (AEs) and complication rates are being evaluated by another part of the presented clinical trial and will be further presented. There were no cases of TT interruption, with most patients suffering from AE not higher than Gr 1 during the treatment period. There were no severe surgical complications in either group according to the Clavien–Dindo scoring system that required repeated intervention during the 90-day postoperative period.

## 4. Discussion

The results of a randomised prospective study evaluating the efficacy of neoadjuvant TT in the treatment of patients with localised RCC showed promising results.

Data from clinical trials indicate a connection between chronic kidney disease and cardiovascular risks. Conservative treatment allows preservation of additional functioning nephrons with reference rates of oncologic survival compared to nephrectomy. Currently, indications for conservative surgical treatment beyond the standard T1a stage are expanding. Particular attention is given to bilateral kidney lesions, single kidney tumours, and RCC with simultaneous opposite kidney pathology, which significantly diminishes its function, while nephrectomy may result in dialysis for such patients. This could induce a problem due to the insufficient number of dialysis beds and their high costs [15]. In these cases, TT aims to reduce the RCC size and increase the likelihood of partial nephrectomy. This appears to be quite attractive from the perspective of RFPV preservation [16, 17].

The principle of neoadjuvant TT in RCC is based on the concept of increasing procedural safety and improving locoregional control [18]. A potential advantage of this approach is its cytoreductive effect and the movement of patients from radical treatment to nephron-sparing surgery. The efficacy of this option was evaluated using sunitinib, sorafenib, pazopanib, and axitinib [19].

The positive effect of neoadjuvant TT was first described by Thomas et al. [12]. The study included 19 patients with advanced RCC ineligible for initial nephrectomy and who were indicated to be treated with sunitinib. Partial response of the primary tumour was observed in 16% (*n* = 3) of patients, stable disease in 37% (*n* = 7), and progression in 47% (*n* = 9). A reduction in tumour size was observed in 8/19 (42%) patients, with an average of 24% reduction, which allowed nephrectomy in four cases.

The role of neoadjuvant TT is even more significant in increasing the likelihood of conservative treatment for RCC. Silberstein et al. demonstrated TT efficacy in a group of 14 tumours (12 patients) with imperative indications, who showed an average reduction in RCC size of 21.1%. In all cases, a partial nephrectomy with a negative surgical margin was performed. No patients required further haemodialysis [8]. These results confirmed the feasibility of using this strategy in highly selective patients to increase the likelihood of organ-sparing surgery.

Another study by Rini et al. showed the feasibility of pazopanib (800 mg) in optimising parenchyma preservation within high-complexity tumours. A common criterion was that the expected eGFR would be less than 30 mL/min/1.73 m^2^ in the case of nephrectomy. In total, 25 patients were enrolled in the study, 71% of whom showed a decrease in the lesion complexity and 92% showed a decrease in the tumour volume after TT. Kidney preservation was eventually performed in 80% (20/25) of cases with an increased probability of parenchyma preservation from 107 to 173 cm^3^ (*p*=0.0015) [10]. Other similar studies by Karam et al. and Lane et al. showed downsizing of primary tumours up to 28.3% and 32%, respectively. A reliable high level of patients responded to axitinib and sunitinib, which eventually allowed conservative surgery [11, 20]. The limitations of these studies were their small cohorts, group heterogeneity, and the absence of randomisation.

The results of the study evaluating the efficacy of neoadjuvant TT in the treatment of localised ccRCC have shown promising results. The high sensitivity of localised RCC to neoadjuvant TT with an objective response of 91% prompted the search for surgical strategy optimisation. A hidden benefit is an increase in the possibility of conservative treatment for complex kidney tumours. This might also lead to a reduction in the incidence of chronic kidney disease in this group and induce life expectancy and quality.

This approach facilitates the selection of surgical tactics for anatomically complex kidney tumours. The neoadjuvant treatment in oncology aims to reduce side effects and improve the tolerability of the selected therapy. This could be explained by the presence of two functioning kidneys, which significantly increased the body's detoxification capabilities.

Our analysis of the correlation between the kidney tumour size before and after neoadjuvant TT using the Pearson method revealed a reliable direct correlation (*r* = 0.77613; *p* < 0.001). We could predict the expected tumour volume after neoadjuvant TT based on the initial tumour size according to tomography (refer to Supplementary Materials). This could help predict possible localised RCC regression and shift tactics to conservative surgical treatment. In contrast, we could conclude on its feasibility (in case of unfavourable results) and prevent unnecessary systemic toxicity.

The dependence of the correlation between the RCC size and regression degree under the influence of neoadjuvant TT was described by Kroon et al. [14]. The authors found a direct correlative dependence based on the treatment of 78 patients with metastatic RCC with different drugs of the group of tyrosine kinase inhibitors. They concluded that the smaller the primary RCC size, the greater the likelihood and more effective the tumour regression. However, the results we obtained did not reveal the dependence of the level of objective tumour response to neoadjuvant TT on the size of the lesion. This could be explained by biological differences within RCC, which could not be evaluated by size.

Considering the above mentioned, it should be noted that neoadjuvant TT in localised ccRCC is a promising method aimed at improving functional outcomes of treatment, thus decreasing the level of patients with further disability. Additionally, the implementation of the technique aims to facilitate the choice of surgical tactics for anatomically complex kidney tumours.

The ongoing study on neoadjuvant TT in localised RCC is aimed at increasing the understanding of anatomical and functional changes in the kidneys affected by TT, evaluation of its efficacy, determination of its indications, prediction of the course of treatment stages, and the choice of surgery type.

## 5. Conclusion

Neoadjuvant TT in patients with surgically complex localised RCC resulted in clinical tumour regression, which provided a higher rate of conservative surgical treatment compared to similar patients without TT.

The positive results of neoadjuvant TT obtained in our study indicate the clinical validity of further investigating preoperative systemic therapy in localised ccRCC.

## Figures and Tables

**Table 1:** Baseline patients characteristics, tumour stage and size, kidney function, and concomitant pathology, *n* = 118.

Indicator	Statistical units	Main group, *n* = 58 (%)	Control group, *n* = 60 (%)	Statistical evaluation
Age, years	M ± SD (95% CI)	55.3 ± 10.3 (52.6–58)	54.5 ± 12 (51.4–57.6)	Mann–Whitney *U* test; *p*=0.8
Sex	Male, *n* (%)	39 (67.2)	34 (56.7)	*x* ^2^ = 1.39 *p*=0.24
	Female, *n* (%)	19 (32.7)	26 (43.3)	
T	1a, *n* (%)	7 (12.1)	5 (8.3)	*x* ^2^ = 7.3; *p*=0.63
	1b, *n* (%)	3 5 (60.3)	31 (51.7)	
	2a, *n* (%)	1 2 (20.7)	22 (36.7)	
	2b, *n* (%)	4 (6.9)	2 (3.3)	

ECOG	Me [25%; 75%]	1 [0, 1]	1 [0, 1]	Mann–Whitney *U* test; *p*=0.63
Body mass index	Me [25%; 75%]	28.6 [25.3; 33.2]	28, 4 [26.9; 30.4]	Mann–Whitney *U* test; *p*=0.64
Haemoglobin	Me [25%; 75%]	130, 5 [115; 141]	135 [118; 149]	Mann–Whitney *U* test; *p*=0.2
Kidney tumour size, mm	M ± SD (95% CI)	60.7 ± 19.8 (55.5–66)	62.5 ± 16.7 (58.2–66.9)	Mann–Whitney *U* test; *p*=0.56
Total glomerular	M ± SD (95% CI)	88.6 ± 26.1(76.7–100.5)	90.5 ± 22.5 (80.5–100.5)	Mann–Whitney *U* test; *p*=0.83
Blood creatinine (*μ*mol/L)	M ± SD (95% CI)	94.5 ± 2 (89.3–99.7)	90 ± 2.4 (83.7–96.5)	Mann–Whitney *U* test; *p*=0.17
CKD	*n* (%)	4 (6, 9)	3 (5)	*x* ^2^ = 0.19; *p*=0.66
Concomitant pathology	*n* (%)	29 (50)	28 (46.7)	*x* ^2^ = 0.13; *p*=0.71

SD, standard deviation; CKD, chronic kidney disease; ECOG, East Cooperation Oncology Group status.

**Table 2:** Distribution of surgical interventions in groups, *n* = 118.

Type of surgical treatment	Main group (neoadjuvant targeted therapy) *n* = 58	Control group (neoadjuvant targeted therapy) *n* = 60	Statistical evaluation
Partial nephrectomy, *n* (%)	53 (91.4)	20 (33.3)	*x* ^2^ = 42.1; *p* < 0.001
Radical nephrectomy, *n* (%)	5 (8.6)	40 (66.7)	

## Data Availability

The database can be provided upon request to the corresponding author via the mail.

## Supplementary Materials

Supplementary Materials Additional Figure 3: comparative assessment of the mean size of localised RCC before and after neoadjuvant TT based on spiral CT data, *n* = 58. Additional Figure 4: correlation of localised RCC size before and after neoadjuvant TT using Pearson correlation analysis. Additional Figure 5: dependence of the localised RCC regression level on the size of the primary tumour in the kidney, *n* = 58. Additional Table 3: RFPV before and after neoadjuvant TT in the main group according to the bolus contrast enhancement spiral CT results, *n* = 58. Additional Table 4: evaluation of objective response according to RECIST 1.1 in localised RCC after neoadjuvant targeted therapy based on spiral CT data, *n* = 58. Additional Table 5: dependence of regression level and tumour stage after neoadjuvant targeted therapy, *n* = 58

## References

[1] NCCN Guidelines Version 2.2019 (2019). Kidney cancer. https://www.nccn.org/professionals/physician_gls/pdf/kidney.pdf.

[2] European Association of Urology (2018). EAU guidelines on renal cell carcinoma 2018 ISBN/EAN: 978-94-92671-01-1. https://uroweb.org/guideline/renal-cell-carcinoma.

[3] Smith D. H., Thorp M. L., Gurwitz J. H. (2013). Chronic kidney disease and outcomes in heart failure with preserved versus reduced ejection fraction. *Circulation: Cardiovascular Quality and Outcomes*.

[4] Go A. S., Chertow G. M., Fan D., McCulloch C. E., Hsu C.-Y. (2004). Chronic kidney disease and the risks of death, cardiovascular events, and hospitalization. *New England Journal of Medicine*.

[5] Herzog C. A., Asinger R. W., Berger A. K. (2011). Cardiovascular disease in chronic kidney disease. A clinical update from Kidney Disease: improving Global Outcomes (KDIGO). *Kidney International*.

[6] Huang W. C., Levey A. S., Serio A. M. (2006). Chronic kidney disease after nephrectomy in patients with renal cortical tumours: a retrospective cohort study. *The Lancet Oncology*.

[7] Powles T., Kayani I., Blank C. (2011). The safety and efficacy of sunitinib before planned nephrectomy in metastatic clear cell renal cancer. *Annals of Oncology*.

[8] Silberstein J. L., Millard F., Mehrazin R. (2010). Feasibility and efficacy of neoadjuvant sunitinib before nephron-sparing surgery. *BJU International*.

[9] Hellenthal N. J., Underwood W., Penetrante R. (2010). Prospective clinical trial of preoperative sunitinib in patients with renal cell carcinoma. *Journal of Urology*.

[10] Rini B. I., Plimack E. R., Takagi T. (2015). A phase II study of pazopanib in patients with localized renal cell carcinoma to optimize preservation of renal parenchyma. *Journal of Urology*.

[11] Lane B. R., Derweesh I. H., Kim H. L. (2015). Presurgical sunitinib reduces tumor size and may facilitate partial nephrectomy in patients with renal cell carcinoma. *Urologic Oncology: Seminars and Original Investigations*.

[12] Thomas A. A., Rini B. I., Lane B. R. (2009). Response of the primary tumor to neoadjuvant sunitinib in patients with advanced renal cell carcinoma. *Journal of Urology*.

[13] Eisenhauer E. A., Therasse P., Bogaerts J. (2009). New response evaluation criteria in solid tumours: revised RECIST guideline (version 1.1). *European Journal of Cancer*.

[14] Kroon B. K., de Bruijn R., Prevoo W., Horenblas S., Powles T., Bex A. (2013). Probability of downsizing primary tumors of renal cell carcinoma by targeted therapies is related to size at presentation. *Urology*.

[15] Shuch B., Linehan W. M., Bratslavsky G. (2011). Repeat partial nephrectomy. *Current Opinion in Urology*.

[16] Stakhovskiy E. O., Voylenko O. A., Vitruk Y. V., Stakhovskiy O. E. (2015). Application of nephrometry for choice of the treatment tactics in patients, suffering nephrocellular cance. *Klin Khir*.

[17] Voylenko O., Vitruk I., Stakhovskyi O. UNCI nephrometry and remaining functional parenchyma as a new tool indicator for partial nephrectomy in RCC.

[18] Bindayi A., Hamilton Z. A., McDonald M. L. (2018). Neoadjuvant therapy for localized and locally advanced renal cell carcinoma. *Urologic Oncology: Seminars and Original Investigations*.

[19] Hutson T. E., Thoreson G. R., Figlin R. A., Rini B. I. (2016). The evolution of systemic therapy in metastatic renal cell carcinoma. *American Society of Clinical Oncology Educational Book*.

[20] Karam J. A., Devine C. E., Urbauer D. L. (2014). Phase 2 trial of neoadjuvant axitinib in patients with locally advanced nonmetastatic clear cell renal cell carcinoma. *European Urology*.

