# Supplementary material for: Efficacy of Neoadjuvant Targeted Therapy in Treatment of Patients with Localised Clear-Cell Renal Cell Carcinoma
Source: Adv Urol. 2021 Apr 30;2021:6674637. doi: 10.1155/2021/6674637 (PMC8105117; doi:10.1155/2021/6674637)
Supplement: Supplementary Materials — Additional Figure 3: comparative assessment of the mean size of localised RCC before and after neoadjuvant TT based on spiral CT data, n = 58. Additional Figure 4: correlation of localised RCC size before and after neoadjuvant TT using Pearson correlation analysis. Additional Figure 5: dependence of the localised RCC regression level on the size of the primary tumour in the kidney, n = 58. Additional Table 3: RFPV before and after neoadjuvant TT in the main group according to the bolus contrast enhancement spiral CT results, n = 58. Additional Table 4: evaluation of objective response according to RECIST 1.1 in localised RCC after neoadjuvant targeted therapy based on spiral CT data, n = 58. Additional Table 5: dependence of regression level and tumour stage after neoadjuvant targeted therapy, n = 58 [file 6674637.f1.zip › 6674637.f1/additional FIGURE 5.pdf]

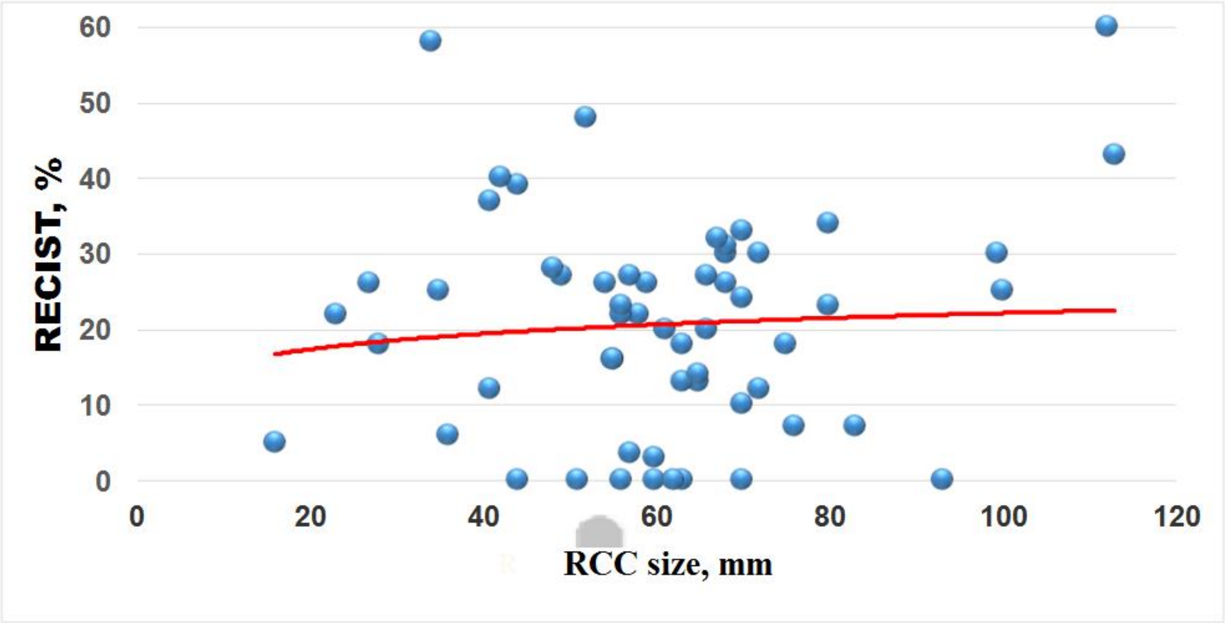

Figure 5 - Dependence of the localized RCC regression level on the size of the primary tumor in the kidney, n = 58
