# Supplementary material for: Efficacy of Neoadjuvant Targeted Therapy in Treatment of Patients with Localised Clear-Cell Renal Cell Carcinoma
Source: Adv Urol. 2021 Apr 30;2021:6674637. doi: 10.1155/2021/6674637 (PMC8105117; doi:10.1155/2021/6674637)
Supplement: Supplementary Materials — Additional Figure 3: comparative assessment of the mean size of localised RCC before and after neoadjuvant TT based on spiral CT data, n = 58. Additional Figure 4: correlation of localised RCC size before and after neoadjuvant TT using Pearson correlation analysis. Additional Figure 5: dependence of the localised RCC regression level on the size of the primary tumour in the kidney, n = 58. Additional Table 3: RFPV before and after neoadjuvant TT in the main group according to the bolus contrast enhancement spiral CT results, n = 58. Additional Table 4: evaluation of objective response according to RECIST 1.1 in localised RCC after neoadjuvant targeted therapy based on spiral CT data, n = 58. Additional Table 5: dependence of regression level and tumour stage after neoadjuvant targeted therapy, n = 58 [file 6674637.f1.zip › 6674637.f1/additional table 5.pdf]

Table 5 - Dependence of regression level and tumor stage, n = 58

T-stage (according to TNM classification)	RCC regression, % M ± SD (95 % CI)	Statistical evaluation
$T_{1a,}$ n = 7	22.9 + 17.7 (6.5 - 39.2)	ANOVA: η^2 = 0.01; power = 0.1; p = 0.72
$T_{1b,}$ n = 35	18.8 +13.2 (14.3 - 23.2)	
$T_{2,}$ n = 16	22.3 + 16.3 (13.6 - 30.9)	
