# Supplementary material for: Efficacy of Neoadjuvant Targeted Therapy in Treatment of Patients with Localised Clear-Cell Renal Cell Carcinoma
Source: Adv Urol. 2021 Apr 30;2021:6674637. doi: 10.1155/2021/6674637 (PMC8105117; doi:10.1155/2021/6674637)
Supplement: Supplementary Materials — Additional Figure 3: comparative assessment of the mean size of localised RCC before and after neoadjuvant TT based on spiral CT data, n = 58. Additional Figure 4: correlation of localised RCC size before and after neoadjuvant TT using Pearson correlation analysis. Additional Figure 5: dependence of the localised RCC regression level on the size of the primary tumour in the kidney, n = 58. Additional Table 3: RFPV before and after neoadjuvant TT in the main group according to the bolus contrast enhancement spiral CT results, n = 58. Additional Table 4: evaluation of objective response according to RECIST 1.1 in localised RCC after neoadjuvant targeted therapy based on spiral CT data, n = 58. Additional Table 5: dependence of regression level and tumour stage after neoadjuvant targeted therapy, n = 58 [file 6674637.f1.zip › 6674637.f1/additonal Table 4 (3) (1).pdf]

Table 4 - Evaluation of objective response according to RECIST 1.1 in localized RCC after neoadjuvant targeted therapy based on spiral CT data, n = 58

Indicator	n (%)	Regression percent median, Me [25%; 75%]
Progression	0	-
Stabile disease	44 (75.9)	16 [5.5; 23.5]
Partial response	14 (24.1)	35.5 [31; 43]
Complete response	0	-
